# Left atrial strain is associated with long-term mortality in acute coronary syndrome patients

**DOI:** 10.1007/s10554-024-03053-7

**Published:** 2024-02-16

**Authors:** Philip Rüssell Pedersson, Kristoffer Grundtvig Skaarup, Mats Christian Højbjerg Lassen, Flemming Javier Olsen, Allan Zeeberg Iversen, Peter Godsk Jørgensen, Tor Biering-Sørensen

**Affiliations:** 1 Department of Cardiology, Copenhagen University Hospital—Herlev & Gentofte, Gentofte Hospitalsvej 8 3Th, Post 835, DK-2900 Copenhagen, Denmark; 2 Center for Translational Cardiology and Pragmatic Randomized Trials, Department of Biomedical Sciences, Faculty of Health and Medical Sciences, University of Copenhagen, Copenhagen, Denmark

**Keywords:** Atrial strain, Speckle tracking echocardiography, Strain imaging, PALS, PCS, PACS

## Abstract

To investigate the long-term prognostic value of the left atrial (LA) strain indices – peak atrial longitudinal strain (PALS), peak conduit strain (PCS), and peak atrial contractile strain (PACS) in acute coronary syndrome (ACS) patients in relation to all-cause mortality. This retrospective study included ACS patients treated with percutaneous coronary intervention (PCI) and examined with echocardiography. Exclusion criteria were non-sinus rhythm during echocardiography, missing images, and inadequate image quality for 2D speckle tracking analysis of the LA. The endpoint was all-cause death. Multivariable Cox regression which included relevant clinical and echocardiographic measures was utilized to assess the relationship between LA strain parameters and all-cause mortality. A total of 371 were included. Mean age was 64 years and 76% were male. Median time to echocardiography was 2 days following PCI. During a median follow-up of 5.7 years, 83 (22.4%) patients died. Following multivariable analysis, PALS (HR 1.04, 1.01–1.06, *p* = 0.002, per 1% decrease) and PCS (HR 1.05, 1.01–1.09, *p* = 0.006, per 1% decrease) remained significantly associated with all-cause mortality. PALS and PCS showed a linear relationship with the outcome whereas PACS was associated with the outcome in a non-linear fashion such that the risk of death increased when PACS < 18.22%. All LA strain parameters remained associated with worse survival rate when restricting analysis to patients with left atrial volume index < 34 ml/m^2^. Reduced LA function as assessed by PALS, PCS, and PACS were associated with an increased risk of long-term mortality in patients with ACS.

**Graphical abstract:**

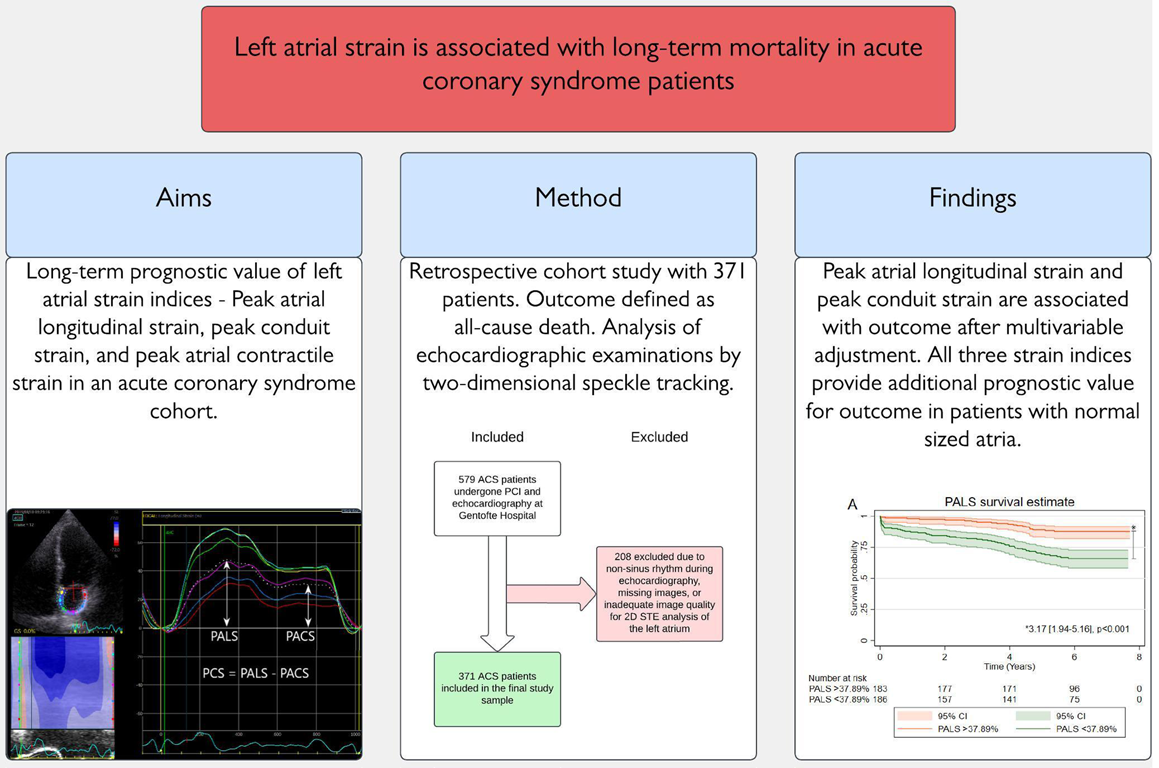

## Introduction

Patients who have suffered from acute coronary syndrome (ACS) are at an increased risk of all-cause mortality, especially over a longer term [1, 2]. Timely identification of patients with an elevated risk of all-cause mortality can provide healthcare professionals with the opportunity to offer early and intensified treatment, potentially improving outcomes, and have socioeconomic benefit [3].

Ongoing efforts to improve the treatment of ACS have yielded positive results over recent decades [4]. This is in large due to improvement in both early invasive management as well as in secondary preventive strategies [5]. Still, in an aging global population with accumulating cardiovascular risk factors, the strain on our healthcare system is expected to rise [6, 7]. It is known that impaired left atrial (LA) function following ACS is associated with major adverse cardiovascular events (MACE) [8]. Risk stratifying ACS patients based upon LA function will assist in identifying patients at high risk of mortality [9]. The non-invasive method of two-dimensional speckle tracking echocardiography (2D STE) provides a detailed assessment of LA function [10].

Previous studies have extensively demonstrated that reduced LA reservoir function assessed by peak atrial longitudinal strain (PALS) is linked to clinical outcomes including all-cause mortality in patients with ST-segment elevation myocardial infarction (STEMI) [11–13] and with acute myocardial infarction (MI) [14]. However, the value of a more comprehensive evaluation of LA function by other strain indices including peak atrial contractile strain (PACS) and peak conduit strain (PCS) in relation to all-cause mortality following ACS remains unknown.

We hypothesized that PALS, PCS, and PACS could be used as prognostic markers for all-cause mortality in patients with ACS. The graphical abstract summarizes aims, methods and main findings of the present study.

## Methods

### Study population

**Fig. 1:**
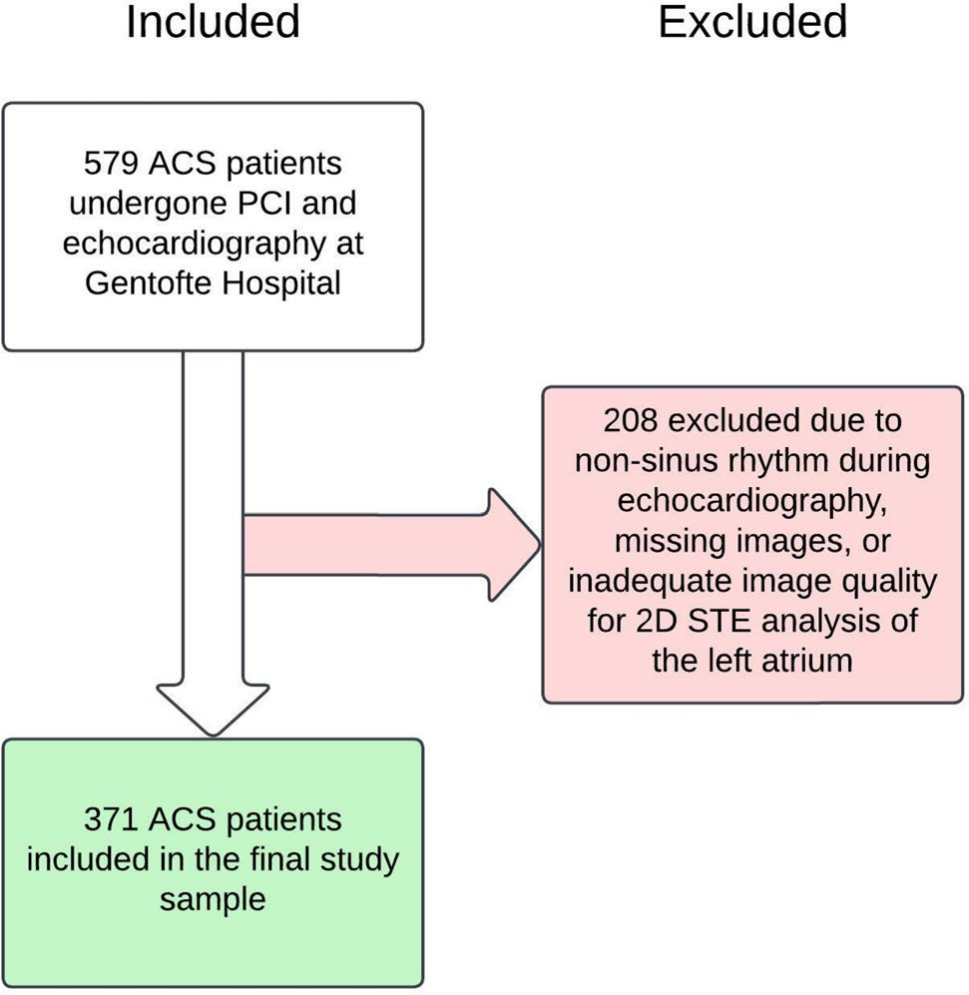
Study population flow diagram. Inclusion and exclusion of patients in study population

During the time period from January 2003 to November 2008, 579 non-consecutive ACS patients were admitted to the Dept. of Cardiology at Gentofte Hospital to have a percutaneous coronary intervention (PCI) performed. These patients were originally part of a larger observational cohort study and described in detail elsewhere [15]. The echocardiographic examinations were performed at Gentofte Hospital a median of 2 days (1–3 days) following the PCI procedure. Screening process for the current study began with 579 ACS patients. Patients were excluded from this study if they had a non-sinus rhythm during echocardiography, missing images, or inadequate image quality for 2D STE analysis. Non-sinus rhythm, which included active atrial fibrillation, was considered an exclusion criterion as PACS would be unobtainable in these patients. Additionally, PALS and PCS would be incomparable between patients with sinus rhythm and those with active atrial fibrillation [16, 17]. A total of 371 non-consecutive ACS patients were included in the final study sample. A flow diagram of the process is illustrated in Fig. 1.

### Grouping

Diabetes mellitus was defined as use of anti-diabetic medicine (oral or injection). Hypertension was defined as use of antihypertensive medication. Hypercholesterolemia was defined as use of cholesterol-lowering medication. Diagnosis of heart failure (HF) was obtained through review of electronic health records at admission.

### Endpoints

Date of PCI designated the beginning of follow-up. Follow-up data on all-cause mortality was retrieved from the Danish National Causes of Death Registry. Endpoint extraction was made in May 2013. Follow-up was complete (100%).

### Echocardiographic examination

Patients underwent transthoracic echocardiography by experienced clinicians and sonographers using GE Vivid ultrasound machines (GE Healthcare, Little Chalfont, UK). The examinations were transferred to and stored on a remote GE Healthcare image archive. All echocardiographic examinations were subsequently analyzed offline including 2D STE using commercially available EchoPac version 202.71 (GE Healthcare, Horten, Norway). The investigator tasked with analysis of the echocardiographic images was blinded to clinical baseline data and endpoints.

### Conventional 2D echocardiography

In the parasternal long axis view at the level of the mitral valve leaflet tips, the left ventricle (LV) dimensions were measured at end-diastole [18]. LV mass index (LVMI) was calculated by dividing the anatomical mass with body surface area (BSA) [18]. LV ejection fraction (LVEF) was measured using the Simpson’s biplane method [18]. LA volume (LAV) was obtained at end-systole in the apical 4- and 2-chamber view by the Simpson’s biplane method [18]. LAV index (LAVI) was acquired by indexing LAV to BSA. Mitral valve inflow at the tip of the mitral valve leaflets was recorded using pulsed-wave Doppler imaging in the apical 4-chamber view to measure the peak velocity blood flow in early diastole (E-wave), peak velocity blood flow in late diastole (A-wave), deceleration time of the early filling (DT), and the E/A ratio. By applying pulsed-wave tissue Doppler imaging with the sample areas placed at the septal and lateral walls of the mitral annulus, early mitral annular diastolic velocity (e’) was measured and the E/e’ ratio was determined [19, 20]. LV diastolic dysfunction was classified based on the E/e’ ratio with values > 14, 9–14 and < 9 considered abnormal, indeterminate, and normal respectively [19]. Right ventricular dysfunction was assessed according to tricuspid annular plane systolic excursion, where < 1.7 cm indicated abnormality [18].

### Speckle tracking echocardiography

**Fig. 2:**
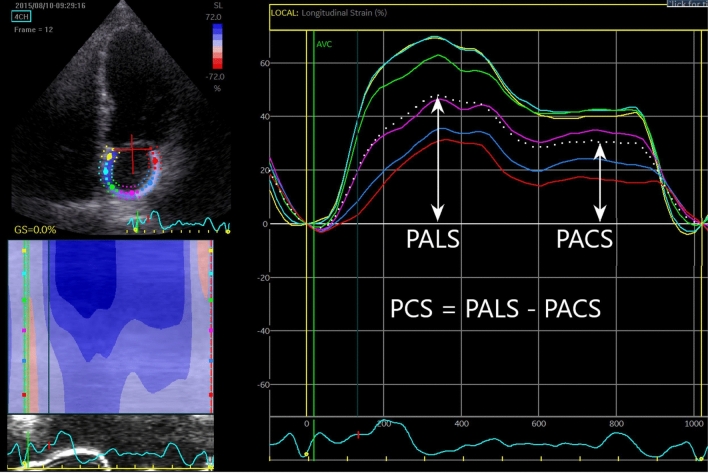
Example of left atrial speckle tracking echocardiography. Example of left atrial speckle tracking from the apical 4-chamber view. Coloured lines represent each segment, and the white dotted line represents the global value

2D STE analysis was performed of the LA in the 4- and 2-chamber views with a manual point and click function that defined a region of interest (ROI) with the option of being adjusted manually by the investigator if the automatic ROI was considered inaccurate. Both the 4- and 2-chamber LA views were divided into 6 segments for a total analysis of 12 segments. If more than two segments were deemed untraceable after manual adjustment, the investigator excluded it from analysis. The following LA strain measures were derived: PALS, PCS, and PACS. The mean frame rate was 71 ± 25. The methodology is illustrated in Fig. 2.

### Statistical analysis

Statistics were carried out using (STATA/SE 17.0). The Wilcoxon rank-sum test was used for comparing continuous non-Gaussian distributed variables, which are presented as interquartile ranges. Pearson’s Chi^2^ test was used for comparing categorical variables, which are expressed as frequencies (percentages). Student’s t-test was used for comparing continuous Gaussian distributed variables, which are displayed as mean values ± standard deviation. All strain values are presented as absolute values. The prognostic value of PALS, PCS, and PACS were assessed by uni- and multivariable Cox proportional hazards regression models. The multivariable model consisted of either PALS, PCS, or PACS and were adjusted for common confounders and clinically significant factors, i.e., age, sex, LVEF, hypertension, diabetes mellitus, heart failure, multivessel disease, global longitudinal strain (GLS), and LAVI. A sensitivity analysis was performed in which the multivariable analyses were restricted to patients with LAVI < 34 mL/m^2^. Reported lower limits of normality in a healthy cohort for PALS, PCS, and PACS were used to calculate sensitivity, specificity, positive predictive value, and negative predictive value for LA strain indices [21]. Poisson regression was used for estimating incidence rates and restricted cubic spline curves were constructed to illustrate the relationship between LA strain indices and the incidence rate of all-cause mortality. The number of optimal knots were determined by the lowest Akaike information criterion. Kaplan–Meier curves were constructed to estimate survival probability by groups of low or high PALS, PCS, and PACS, defined by median values. A *p*-value < 0.05 was considered statistically significant in two-tailed tests.

## Results

The final study population consisted of 371 ACS patients following exclusions. Time from onset of ACS to post-PCI echocardiography was median 2 (IQR 1–3) days. The mean age was 64 ± 12 years, and the population predominantly consisted of males (76%). During a median follow-up time of 5.7 (IQR 4.7–6.9) years, 83 (22.4%) patients died. Median time to death in non-survivors was 252 (IQR 44–668) days. Table 1 displays baseline characteristics stratified according to outcome.

**Table 1:** Baseline and clinical characteristics stratified according to endpoint

Variable	All	Alive	Deceased	*P* value for trend
*Demographics*				
Number of patients	371	288	83	
Age, years	63.6 ± 12.2	60.8 ± 10.9	73.2 ± 11.8	< *0.001*
Male sex, *n* (%)	280 (75.5)	230 (79.9)	50 (60.2)	< *0.001*
*Clinical characteristics*				
Systolic blood pressure, mmHg	136.8 ± 26.3	137.2 ± 25.9	135.3 ± 27.8	*0.56*
Diastolic blood pressure, mmHg	81.7 ± 16.4	82.2 ± 15.3	79.7 ± 19.6	*0.23*
Heart rate, beats per minute	73.9 ± 14.7	72.4 ± 13.6	79.3 ± 17.2	< *0.001*
Use of antihypertensive medication, *n* (%)	230 (62.0)	192 (66.7)	38 (45.8)	< *0.001*
Use of cholesterol-lowering medication, *n* (%)	82 (22.1)	62 (21.5)	20 (24.1)	*0.62*
Active smoker, *n* (%)	171 (67.9)	141 (71.2)	30 (55.6)	*0.039*
Body mass index, kg/m^2^	26.3 ± 4.3	26.7 ± 4.1	25.1 ± 4.6	*0.003*
Diabetes mellitus, *n* (%)	31 (8.4)	21 (7.3)	10 (12.0)	*0.17*
History of heart failure, *n* (%)	16 (4.3)	7 (2.4)	9 (10.8)	< *0.001*
Family history of CVD, *n* (%)	115 (31)	95 (33)	20 (24)	*0.12*
*Acute coronary syndrome information*				
STEMI, *n* (%)	285 (76.8)	219 (76.0)	66 (79.5)	*0.51*
Multivessel lesion, *n* (%)	25 (6.7)	18 (6.2)	7 (8.4)	*0.48*
Vessel treated, *n* (%)				*0.012*
CX	53 (14.3)	43 (14.9)	10 (12.0)	
LAD	184 (49.6)	142 (49.3)	42 (50.6)	
LMS	3 (0.8)	0 (0.0)	3 (3.6)	
RCA	131 (35.3)	103 (35.8)	28 (33.7)	

Data is depicted as mean ± SD, median with IQR, and total numbers and proportions for normal distributed, non-normal distributed and categorical variables respectively

*CVD* cardiovascular disease, *STEMI–ST*-segment elevation myocardial infarction, *PCI* percutaneous coronary intervention, *CX* circumflex artery, *LAD* left anterior descending artery, *LMS* left main stem coronary artery, *RCA* right coronary artery

Those who died were older (73.2 vs 60.8 years, *p* < 0.001), had higher heart rate (79.3 ± 17.2 vs 72.4 ± 13.6, *p* < 0.001), suffered less frequently from hypertension (45.8 vs 66.7%, *p* < 0.001), fewer were active smokers (55.6 vs 71.2%, *p* = 0.039), and had a lower body mass index (25.1 ± 4.6 vs 26.7 ± 4.1, *p* = 0.003). Looking at the echocardiographic measures shown in Table 2, non-survivors had significantly lower LVEF (35.5 vs 42.6% *p* < 0.001), PALS (30.5 vs 42.9%, *p* < 0.001), PACS (16.5 vs 20.6%, *p* < 0.001), PCS (14.9 vs 22.6%, *p* < 0.001), and GLS (10.7 vs 13.4%, *p* < 0.001). Table 3 lists all Cox regression models performed.

**Table 2:** – Echocardiographic characteristics stratified according to endpoint

Variable	All	Alive	Deceased	*P* value for trend
*Echocardiographic measures*				
TAPSE cm	1.9 ± 0.4	1.9 ± 0.4	1.7 ± 0.4	< *0.001*
< 1.7 cm, *n* (%)	109 (29.4)	70 (24.3)	39 (47.0)	
≥ 1.7 cm, *n* (%)	262 (70.6)	218 (75.7)	44 (53.0)	
TIMI Grade pre-PCI, *n* (%)				*0.516*
0	83 (57.2)	64 (59.8)	19 (50.0)	
1	24 (16.6)	16 (15.0)	8 (21.1)	
2	16 (11.0)	12 (11.2)	4 (10.5)	
3	22 (15.2)	15 (14.0)	7 (18.4)	
TIMI Grade post-PCI, *n* (%)				*0.568*
0	6 (4.1)	4 (3.7)	2 (5.3)	
1	4 (2.8)	2 (1.9)	2 (5.3)	
2	9 (6.2)	7 (6.5)	2 (5.3)	
3	126 (86.9)	94 (87.9)	32 (84.2)	
IVSd cm	1.1 ± 0.7	1.1 ± 0.2	1.2 ± 1.4	*0.092*
LVIDd cm	5.0 ± 2.4	4.9 ± 0.5	5.4 ± 5.2	*0.17*
LVPWd cm	1.0 ± 0.6	1.0 ± 0.2	1.1 ± 1.2	*0.14*
LVMi g/m^2^	89.5 [76.6, 103.9]	90.2 [76.2, 104.5]	86.1 [77.5, 99.4]	*0.34*
LVEF (%)	41.0 ± 11.4	42.6 ± 10.4	35.5 ± 12.8	< *0.001*
≤ 40%, *n* (%)	159 (42.9)	108 (37.5)	51 (61.5)	
41–49%, *n* (%)	122 (32.9)	106 (36.8)	16 (19.3)	
≥ 50%, *n* (%)	90 (24.3)	74 (25.7)	16 (19.3)	
E/A-ratio	1.0 ± 0.4	1.0 ± 0.3	1.0 ± 0.5	*0.35*
E/e'	9.6 [7.7, 12.1]	9.2 [7.5, 11.4]	11.3 [8.7, 14.6]	< *0.001*
< 9, *n* (%)	143 (42.9)	122 (46.4)	21 (30.0)	
9–14, *n* (%)	138 (41.4)	110 (41.8)	28 (40.0)	
> 14, *n* (%)	52 (15.6)	31 (11.8)	21 (30.0)	
LAVI mL/m^2^	25.1 ± 8.9	24.8 ± 8.9	25.9 ± 8.7	*0.34*
GLS (%)	12.8 ± 4.0	13.4 ± 3.6	10.7 ± 4.4	< *0.001*
PALS (%)	40.1 ± 17.1	42.9 ± 17.0	30.5 ± 13.7	< *0.001*
PACS (%)	19.7 ± 9.8	20.6 ± 9.7	16.5 ± 9.4	< *0.001*
PCS (%)	21.0 ± 10.7	22.6 ± 10.9	14.9 ± 7.1	< *0.001*

Data is depicted as mean ± SD, median with IQR, and total numbers and proportions for normal distributed, non-normal distributed and categorical variables respectively

*TAPSE* tricuspid annular plane systolic excursion, *TIMI* thrombolysis in myocardial infarction, *IVSd* interventricular septal end diastole, *LVIDd* left ventricular internal diameter, *LVPWd* left ventricular posterior end diastole, *LVMi* left ventricular mass indexed to body surface area, *LVEF* left ventricular ejection fraction, *LAVI* left atrial volume index, *GLS* global longitudinal strain, *PALS* peak atrial longitudinal strain, *PACS* peak atrial contractile strain, *PCS* peak conduit strain

**Table 3:** Cox regression for univariable and multivariables stratified for endpoint all-cause death

	Hazard ratio	95% CI	*P*-value
*Univariable*			
PCS	1.12	1.08–1.15	< 0.001
PACS	1.05	1.02–1.08	< 0.001
PALS	1.06	1.04–1.08	< 0.001
*Multivariable model*			
PCS	1.05	1.01–1.09	0.006
PACS	1.03	0.99–1.06	0.094
PALS	1.04	1.01–1.06	0.002
PACS < 18.22%	1.61	0.95–2.74	0.079
*Sensitivity analysis*			
PCS	1.05	1.01–1.09	0.023
PACS	1.04	1.01–1.08	0.019
PALS	1.04	1.02–1.07	0.001
PCS < 18.75%	1.40	0.77–2.53	0.265
PACS < 18.22%	1.89	1.08–3.31	0.026
PALS < 37.89%	2.12	1.18–3.79	0.011

All variables are expressed as per 1% decrease. Multivariable model included adjustments for age, sex, left ventricular ejection fraction, hypertension, diabetes mellitus, heart failure, culprit lesion, global longitudinal strain, and LAVI. The sensitivity analysis utilized the same multivariable model, but was restricted to patients with LAVI smaller than 34 ml/m^2^

*PALS* peak atrial longitudinal strain, *PACS* peak atrial contractile strain, *PCS* peak conduit strain, *CI* confidence interval

**Fig. 3:**
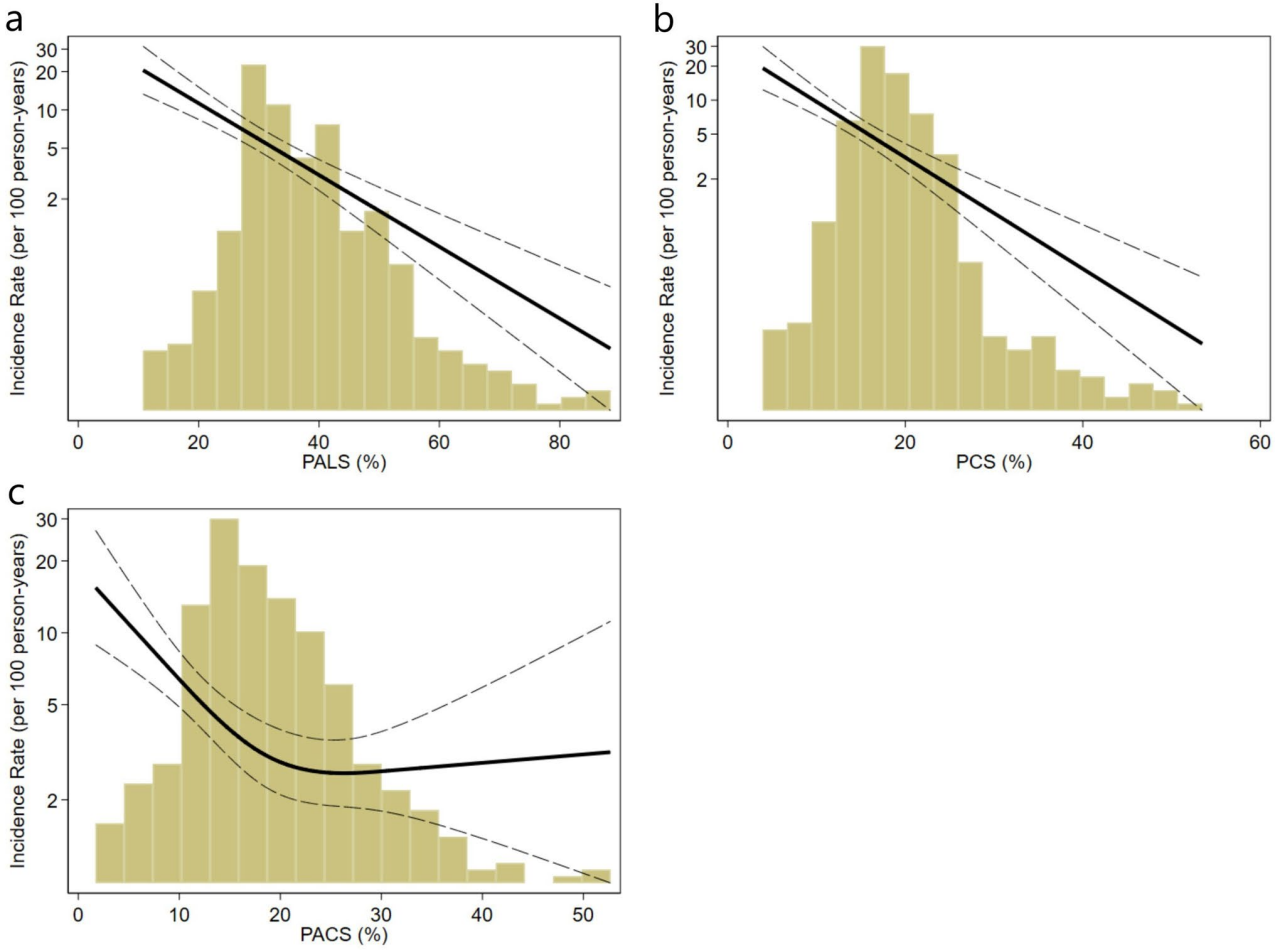
Risk of dying by continuous changes in left atrial strain indices. Restricted cubic spline curves displaying the unadjusted incidence rate of all-cause death per 100 patient-years as a function of the three LA strain measures, thus illustrating the risk of dying with decreasing values for **A** PALS. **B** PCS. **C** PACS

PALS, PCS, and PACS (PALS: HR 1.06, 1.04–1.08, *p* < 0.001, per 1% decrease; PCS: HR 1.12, 1.08–1.15, *p* < 0.001, per 1% decrease; PACS: HR 1.05, 1.02–1.08, *p* < 0.001, per 1% decrease) were all significantly associated with all-cause mortality in univariable regressions. In multivariable adjustment, only PCS and PALS (PCS: HR 1.05, 1.01–1.09, *p* = 0.006, per 1% decrease; PALS: HR 1.04, 1.01–1.06, *p* = 0.002, per 1% decrease) remained significantly associated with all-cause mortality. The continuous relationships between LA strain measures and all-cause mortality are illustrated in Fig. 3a–c.

**Fig. 4:**
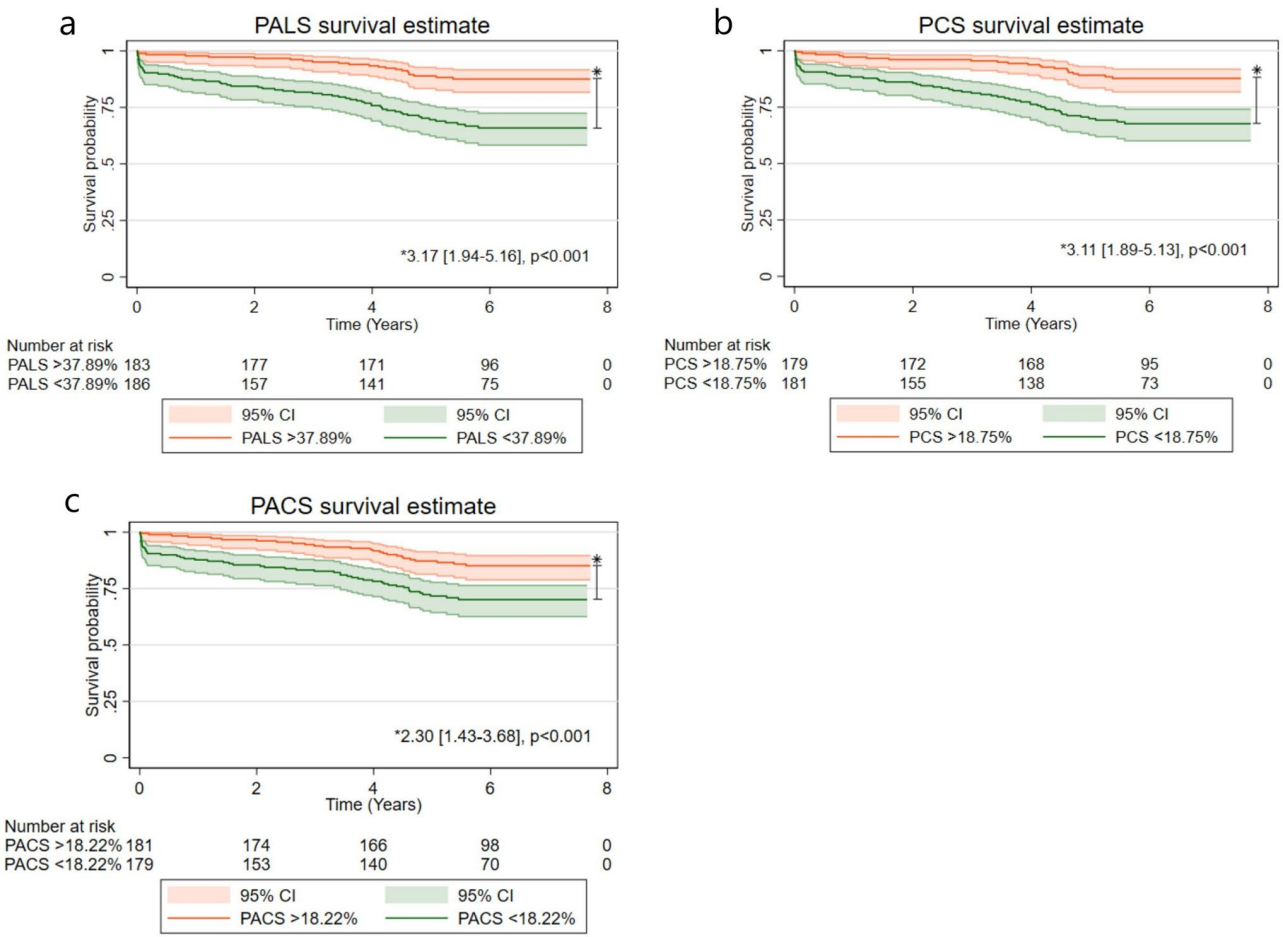
Kaplan–Meier estimators stratified according to abnormal LA strain values. Kaplan–Meier curves displaying probability of staying alive throughout the follow-up period. The x-axis displays the time from exposure (ACS). The y-axis represents the cumulative probability of survival. The study population is stratified into two groups based on whether they are above or below the median of the LA strain measurements (**A** PALS. **B** PCS. **C** PACS). The star symbol denotes the HR

PALS and PCS showed a linear relationship with all-cause mortality. PACS showed a non-linear relationship such that the incidence rate of death did not increase before PACS decreased below approximately 18.22% corresponding to the median value. The survival probabilities according to medians of PALS, PCS, and PACS are illustrated in Kaplan Meier curves in Fig. 4a–c. All three LA strain indices were significantly associated with all-cause death when the analysis was restricted to individuals with normal LAVI in the multivariable model, (PALS: HR 1.04, 1.02–1.07, *p* = 0.001, per 1% decrease; PCS: HR 1.05, 1.01–1.09, *p* = 0.023, per 1% decrease; PACS: HR 1.04, 1.01–1.08, *p* = 0.019, per 1% decrease). Finally, when restricting the analysis to patients with LAVI < 34 mL/m^2^, with LA strain indices below its median PALS and PACS remained significantly associated with all-cause death (PALS < 37.89%: HR 2.12, 1.18–3.79, *p* = 0.011, per 1% decrease; PCS < 18.75%: HR 1.40, 0.77–2.53, *p* = 0.265, per 1% decrease; PACS < 18.22%: HR 1.89, 1.08–3.31, *p* = 0.026).

The sensitivity, specificity, positive predictive value, and negative predictive value according to reported lower limits of normality for PALS (< 23%), PCS (< 8.8%), and PACS (< 6.4%) are listed in Table 4.

**Table 4:** Sensitivity, specificity, positive predictive value, and negative predictive value

Variable	Sensitivity (%)	Specificity (%)	PPV (%)	NPV (%)
PALS	28.9	94.8	61.5	82.2
PCS	19.3	96.2	59.3	80.5
PACS	8.43	96.2	38.9	78.5

*PALS* peak atrial longitudinal strain, *PACS* peak atrial contractile strain, *PCS* peak conduit strain, *PPV* positive predictive value, *NPV* negative predictive value

## Discussion

In the present study, we examined the prognostic value of the LA strain indices PALS, PCS, and PACS in regards to long-term survival rate in ACS patients who had echocardiography performed median 2 days after PCI. We made several significant findings: (1) Continuously decreasing PALS and PCS were associated with increased risk of death following multivariable adjustments. (2) PACS was associated with the outcome in a non-linear fashion in patients with normal sized LA such that the risk of death only increased when PACS < 18.22%. (3) All parameters remained significantly associated with all-cause mortality when restricting analysis to patients with normal LAVI.

Previous studies have evaluated the prognostic value of PALS in relation to all-cause death in ACS cohorts. This study was the first to investigate the prognostic value of the additional strain measurements PACS and PCS in an ACS population. A study consisting of 320 STEMI patients treated with PCI achieved results similar to ours. It found PALS as an independent predictor of a composite endpoint consisting of all-cause death, reinfarction and hospitalization due to HF, where 48 (15%) patients reached the combined endpoint [12]. This contrasts with Ersbøll et al. [14], who conducted a study in an MI cohort of 843 patients with a composite endpoint including all-cause death and HF hospitalization in which 47 (5.6%) and 29 (3.4%) patients reached these outcomes, respectively. Echocardiography was performed within 48 h of admission to tertiary hospital where 79.6% of admitted patients underwent PCI. The authors found PALS to be a univariable predictor of outcome, but it was insignificant when adjusting for GLS, age, and LAV, thus concluding that PALS did not provide additional prognostic information over conventional measures. The discrepancy in findings between Ersbøll et al. and the present study may be explained by the difference in endpoints (all-cause mortality vs all-cause mortality and HF hospitalizations) or differences in baseline clinical characteristics. The present cohort, compared to Ersbøll et al. was older, had lower LVEF, and had a higher frequency of hypertension and STEMI indicating that Ersbøll et al. investigated a healthier cohort compared to ours. Such differences could explain the discrepancy in the results. Furthermore, a recent study of patients with HF with the same composite outcome as Ersbøll et al. reported results similar to ours. They found PALS to be a strong prognostic marker in 405 stable HF patients with LVEF < 40%, independent of both GLS and LAV [22]. The three different studies all evaluated the association between PALS and outcome. In summary, they found PALS to be an univariable predictor of outcome and in the majority of studies, it was still significant when accounting for GLS and LAV. Unfortunately, the number of studies investigating the prognostic ability of PCS and PACS is limited and no study has previously investigated the prognostic value of PCS and PACS regarding mortality outcomes among patients with ischemic heart disease. However, a study by Li et al. [9] of 229 ACS patients examined by echocardiography median of 1 day before PCI, demonstrated PALS to have the strongest correlation with global registry of acute coronary events (GRACE) score out of all investigated echocardiographic variables (which included a vast number of structural and functional echocardiographic measurements), closely followed by PCS and PACS. Finally, Svartstein et al. [23] examined 392 STEMI patients following PCI and observed decreasing PALS, PCS, and PACS to be associated with incident atrial fibrillation. However, only PALS remained significant after multivariable analysis. Although these studies are not directly comparable to the present study due to the different investigated endpoints, their observation of similar patterns aligns with our own findings.

In this study, we found a significant association between continuously decreasing PALS and PCS, and an increased risk of death. In contrast, we found that PACS was not significantly associated with an increased risk of death until levels were below 18.22%. This discrepancy may be attributed to the distinct pathophysiological development paths of PALS, PCS, and PACS. PALS and PCS tend to develop in a unidirectional manner, while PACS can exhibit a bidirectional pathophysiological progression [24]. In general, the interdependent LA measurements PALS, PCS, and PACS represent reservoir phase, conduit phase, and contractile phase of the LA respectively [25]. Reservoir phase for the pulmonary venous return is primarily influenced by longitudinal displacement of the LV during contraction and LA myocardial compliance. Conduit phase reflects LV relaxation translated as the passive flow of blood from LA to LV. Contractile phase relies on LV filling pressures and synchronous electromechanical activity. Increased LV filling pressure is common after myocardial infarction and leads to reduced passive filling of the LV from the LA. This is reflected by a reduction in PCS. The LA will compensate by increasing its contractile pressure thereby maintaining PACS. The increase in LA contractile pressure causes reflux to the pulmonary veins leading to increased LA preload which can result in LA remodeling and subsequent decrease in PACS [26]. Persistently increased LV filling pressure can cause LA remodeling and subsequent impaired LA contractile function, which will be observed as a decrease in PACS.

LA strain is potentially a better indicator of LA function and prognosis following ACS than LAVI (the only recommended parameter to evaluate). The study findings indicate that LA strain parameters may provide valuable additional prognostic information in identifying individuals at a higher risk of early death following ACS, even when restricted to normal LAVI. Patients with PALS and PCS below the study median had a more than threefold higher risk of dying than patients above the median. Furthermore, in patients without LA remodeling assessed by LAVI, all LA strain parameters provided long-term prognostic value. Studies have found LA strain to yield predictive value for development of complications associated with increased mortality such as MACE, atrial fibrillation and HF in ACS cohorts [11, 23, 27]. The previously mentioned study by Li et al. [9]*.* found PALS to be superior to LAVI by comparing their correlation with GRACE risk scores in predicting short term MACE [18, 28, 29]. Furthermore, accurate determination of LAVI has shown to be difficult in under-/overweight patients, and deterioration of LA function can occur before an increase in LAVI [30–32]. Inclusion of LA strain analysis in the echocardiographic examination post-PCI is time efficient and simple and may be helpful in detection of high-risk patients. This has the potential to assist in identifying individuals who require intensified follow-up, monitoring, and potential risk intervention, as well as those at a lower risk who may need less frequent monitoring. However, it is crucial to note that these findings should be considered hypothesis-generating, and definitive conclusions would necessitate larger prospective studies or randomized controlled trials.

## Limitations

This study has important limitations that must be acknowledged. Firstly, the analysis of LA speckle tracking was performed using non-dedicated software that was originally developed for the LV. This, along with the reliance on vendor-dependent software, may limit the generalizability of our results to other study samples that were analyzed using different echocardiographic software and hardware [33]. However, Mirea et al. [34] compared the use of LV and dedicated LA tracking tools for measuring LA strain, and found no statistical difference in strain value. Additionally, as LA dedicated automatic software was unavailable at the time of analysis, we were unable to perform intra- and inter-observer analysis between LA strain measured by the method used in the present study and LA dedicated automatic software, Secondly, in this study, we adhered to the currently recommended method of measuring LAVI using 2D images [35]. However, the possibility of underestimating true LA volumes by using 2D STE over 3D cannot be dismissed. Thirdly, due to the retrospective nature of the study, several potentially residual confounders related to additional pharmacological treatment, relevant chronic conditions such as paroxysmal/persistent atrial fibrillation, mitral regurgitation, history of important events—e.g., stroke—and certain biochemical markers including estimated glomerular filtration rate, creatine phosphokinase, brain natriuretic peptide, troponin, and reperfusion status were unavailable. Moreover, the potential impact of undiagnosed pre-existing conditions in patients prior to their initial hospitalization for ACS cannot be ruled out, which may have influenced our results. Lastly, since the study sample primarily comprised individuals of Scandinavian descent, caution should be exercised when extrapolating the findings to other ethnicities.

## Conclusion

In patients with ACS, reduced LA function by lower PALS, PCS, and PACS were in univariable analysis associated with an increased risk of long-term mortality. Impaired PALS and PCS remained associated with mortality following multivariable adjustments. Lastly, PALS, PCS, and PACS provided prognostic value in patients with normal-sized LA.

## Data Availability

The data underlying this article cannot be shared publicly due to Danish and European data laws. The data will be shared on reasonable request to the corresponding author.
